# Usefulness of Decrease in Oxygen Uptake Efficiency to Identify Gas Exchange Abnormality in Patients with Idiopathic Pulmonary Arterial Hypertension

**DOI:** 10.1371/journal.pone.0098889

**Published:** 2014-06-06

**Authors:** Xiaoyue Tan, Wenlan Yang, Jian Guo, Yan Zhang, Changwei Wu, Rikesh Sapkota, Shailendra Prasad Kushwaha, Sugang Gong, Xingguo Sun, Jinming Liu

**Affiliations:** 1 Department of Pulmonary Function Test, Shanghai Pulmonary Hospital Affiliated to Tongji University, Shanghai, China; 2 Respiratory Division, Hangzhou, Zhejiang Provincial Hospital of traditional Chinese Medicine, The First Affiliated Hospital of Zhejiang Chinese Medicine University, Hangzhou, China; 3 Department of Pulmonary Circulation, Shanghai Pulmonary Hospital Affiliated to Tongji University, Shanghai, China; 4 State Key Laboratory of Cardiovascular Disease, Heart-Lung Function Testing Center, Fuwai Hospital, National Center for Cardiovascular Diseases, Chinese Academy of Medical Sciences and Peking Union Medical College, Beijing, China; Vanderbilt University Medical Center, United States of America

## Abstract

**Background:**

Decline in oxygen uptake efficiency (OUE), especially during exercise, is found in patients with chronic heart failure. In this study we aimed to test the validity and usefulness of OUE in evaluating gas exchange abnormality of patients with idiopathic pulmonary arterial hypertension (IPAH).

**Methods:**

We retrospectively investigated the cardiopulmonary exercise test (CPET) with gas exchange measurements in 32 patients with confirmed IPAH. All patients also had resting hemodynamic measurements and pulmonary function test (PFT). Sixteen healthy subjects, matched by age, sex, and body size were used as controls, also had CPET and PFT measurements.

**Results:**

In IPAH patients, the magnitude of absolute and percentage of predicted (%pred) oxygen uptake efficiency slope (OUES) and oxygen uptake efficiency plateau (OUEP), as well as several other CPET parameters, were strikingly worse than healthy subjects (*P*<0.0001). Pattern of changes in OUE in patients is similar to that in controls, In IPAH patients, OUE values at rest, warming up, anaerobic threshold and peak exercise were all significantly lower than in normal (*P*<0.0001). OUEP%pred, better than OUES%pred, correlated significantly with New York Heart Association (NYHA) functional Class (*r* = −0.724, *P*<0.005), Total Pulmonary Vascular Resistance (TPVR) (*r* = −0.694, *P*<0.005), diffusing capacity for carbon monoxide (DL_CO_) (*r* = 0.577, *P*<0.05), and the lowest ventilation versus CO_2_ output ratio during exercise (LowestV˙E/V˙CO_2_) (*r* = −0.902, *P*<0.0001). In addition, the coefficient of variation (COV) of OUEP was lower (20.9%) markedly than OUES (34.3%) (*P*<0.0001).

**Conclusions:**

In patients with IPAH, OUES and OUEP are both significantly lower than the healthy subjects. OUEP is a better physiological parameter than OUES in evaluating the gas exchange abnormality of patients with IPAH.

## Introduction

Idiopathic pulmonary arterial hypertension (IPAH) is a progressive and fatal disease caused by pulmonary vasculopathy [1]–[2]. Low perfusion to the lungs due to inability of the right ventricle to adequately increase pulmonary blood flow (cardiac output [CO]) for O_2_ exercise demand, gives rise to mismatching of ventilation/perfusion (V/Q) and inefficient lung gas exchange. Cardiopulmonary exercise test (CPET) with gas exchange measurements has proved to be a powerful tool to detect abnormalities in patients with IPAH during exercise [3]. Patients with IPAH can safely undergo noninvasive cycle ergometer testing to their maximal tolerance [4]. The key CPET characteristics in these patients include a diminished aerobic capacity, an impaired ventilatory efficiency and a decreased minute O_2_ uptake versus heart rate at peak exercise (peak V˙O_2_/HR) etc [4]–[8]. These CPET parameters have been widely utilized to grade the severity of exercise limitation, to detect exercise-induced right-to-left shunting, to assess responses to therapy, and to predict prognosis in IPAH patients [5]–[8]. Because of the increasing recognition of potential value of CPET in patients with IPAH, more CPET indexes are required in clinical practice.

Oxygen uptake efficiency (OUE) is a recently emerging parameter which is not obvious in the traditional Wasserman CPET 9-panel plots [9]. OUE measures the change in minute oxygen uptake (V˙O_2_) relative to minute ventilation (V˙E). The most widely studied index of OUE is the oxygen uptake efficiency slope (OUES), which ordinarily mathematically describes a near-linear relationship for V˙O_2_ versus V˙E after transforming V˙E from a linear to log scale. Thus OUES defines the slope of V˙O_2_-vs-logV˙E during an entire exercise period [10]. It was used initially in young patients (mean age 12 yrs) with cardiac disease and then later in adults with heart disease to assess exercise capacity, severity and prognosis [11]–[13]. Recently, the other index of OUE, oxygen uptake efficiency plateau (OUEP) was added. It is well known that the relationship between V˙O_2_ and V˙E during an incremental exercise test is curvilinear due to hyperventilation stimulated by the excess [H^+^] of the acidosis of heavy exercise [14]. We found that V˙O_2_/V˙E when plotted against time normally reached its highest and briefly stable values (plateau) near the anaerobic threshold (AT), before declining due to hyperventilation stimulated by the metabolic acidosis [14]. We defined the highest 90 sec average of V˙O_2_/V˙E as OUEP. In our CHF patients and normal subjects, we found that the OUEP had less variability and higher predictability and test-retest reproducibility than the OUES. It follows that OUEP may have the potential to better assess severity of dysfunction and to better prognosticate mortality and morbidity in patients with either chronic left or right heart failure [15].However, previously we did not investigate this issue in our patients with pulmonary hypertension .

OUE representing the change in V˙_2_ as related to V˙E, could be affected by cardiac output (CO), difference between systemic and pulmonary arterial blood O_2_ contents, lung gas exchange, and changes in pH. OUES is the most widely studied index of OUE, but OUEP, which has not been previously studied in IPAH patients, may have advantages. We had already shown V˙O_2_/V˙E is lower and can decline in the transition from rest to exercise in patients with left heart failure [15]–[16]. We hypothesized that OUEP in IPAH patients could be lower than the normals and decline in the transition from rest to exercise due to inability to adequately increase cardiac output during exercise.

## Methods

### Patients and control subjects

We retrospectively investigated the exercise pathophysiology in 32 patients with IPAH referred for evaluation and treatment in Shanghai Pulmonary Hospital between 2009 and 2012. For comparison purposes, the CPET and pulmonary function test (PFT) data of 16 healthy subjects of similar age, sex, and body size were also analyzed (6 men and 10 women; mean age 37.88±16.76 yrs). All CPET study participants signed written informed consent. This study was approved by the Institution of Human Subjects Committee at Shanghai Pulmonary Hospital. The diagnosis of IPAH was based on clinical and laboratory data, including right heart catheterization (RHC), according to currently accepted diagnostic criteria (Dana Point, 2008) [17]. Patients with disorders other than IPAH were excluded according to the recommended diagnostic guidelines for IPAH [17]. The patients were non-smokers at the time of study and most had never smoked. The data included only the first PFT and CPET measurements made after referral to our hospital, nearly always prior to the initiation of pulmonary vasodilator therapy.

### PFT Measurements

Each patient and normal subject underwent resting measurements of forced vital capacity (FVC), forced expiratory volume in 1 sec (FEV_1_), maximum voluntary ventilation (MVV), diffusing capacity for carbon monoxide (DL_CO_) and total lung capacity (TLC) using standard methodology[18]–[19] and equipments (Masterscreen-PFT, Jaeger, Hoechberg, Germany; Masterscreen-plethysmography, Jaeger, Hoechberg, Germany). All PFT values were reported in absolute terms and normalized to percentage of predicted (%pred). Predicted spirometry values, TLC and DL_CO_ were calculated using accepted equations for Chinese adults [20].

### CPET Procedure and Data Collection

Each patient performed PFT and CPET, after familiarization with the exercise apparatus, on the same day. Before each test, the equipment was calibrated according to manufacturer's specifications using reference and calibration gases. Standard 12 lead electrocardiograms (ECGs) and pulse oximetry were continuously monitored. Blood pressure at the brachial artery was measured every two minutes with an automatic cuff. The exercise protocol consisted of 3 min of rest, 3 min of unloaded cycling at 60 rpm, followed by uniform increase in resistance of 5 to 15 W/min for the patients and 20 to 25 W/min for the normal subjects to maximal tolerance on an electromagnetically braked cycle ergometer (Ergoselect 100, ergoline GmbH, Bitz, Germany) [9]. The rate of increasing work depended on the estimated exercise capacity of the subjects. Subjects were encouraged to exercise to the limits of their functional capacities or until the physician stopped the test because of severe adverse events, such as chest pain, light-headedness, potentially life-threatening arrhythmias, ST-segment changes, or marked systolic hypotension. Most CPET values were reported in absolute terms and normalized to percentage of predicted (%pred). Predicted values were calculated using accepted equations [9].

### CPET data Calculations

Carbon dioxide output (V˙CO_2_, ml/min, STPD), V˙O_2_ (ml/min, STPD), V˙E (l/min, BTPS), tidal volume (l, BTPS), were measured continuously on a breath-by-breath basis using a CPX Metabolic Measurement Cart (Masterscreen-CPX, Jaeger, Hoechberg, Germany) that was equipped with rapidly-responding O_2_ and CO_2_ analyzers. Data were averaged every 10 sec. Peak V˙O_2_ was defined as the highest 30 sec average of V˙O_2_, and other peak parameters were calculated at the same time. Each AT was determined by the V-slope method [21]. V˙E-V˙CO_2_ slope was determined by linear regression analysis of the relation between V˙E and V˙CO_2_ during exercise, excluding data above the ventilatory compensation point [22]. Lowest V˙E/V˙CO_2_ was determined by averaging the lowest consecutive 90 sec data points [22].

In addition, to show patterns of gas exchange change in patients as related to time and exercise intensity during CPET, V˙O_2_/V˙E, V˙E/V˙CO_2_ and P_ET_CO_2_ values at 4 periods were respectively averaged: the last minute of rest, last minute of unloaded cycling, 1 min before the AT was reached (only for V˙O_2_/V˙E), 1 min after the AT was reached (only for V˙E/V˙CO_2_ and P_ET_CO_2_) and for 30 sec at peak exercise.

### OUE definitions and measurements

**Figure 1:**
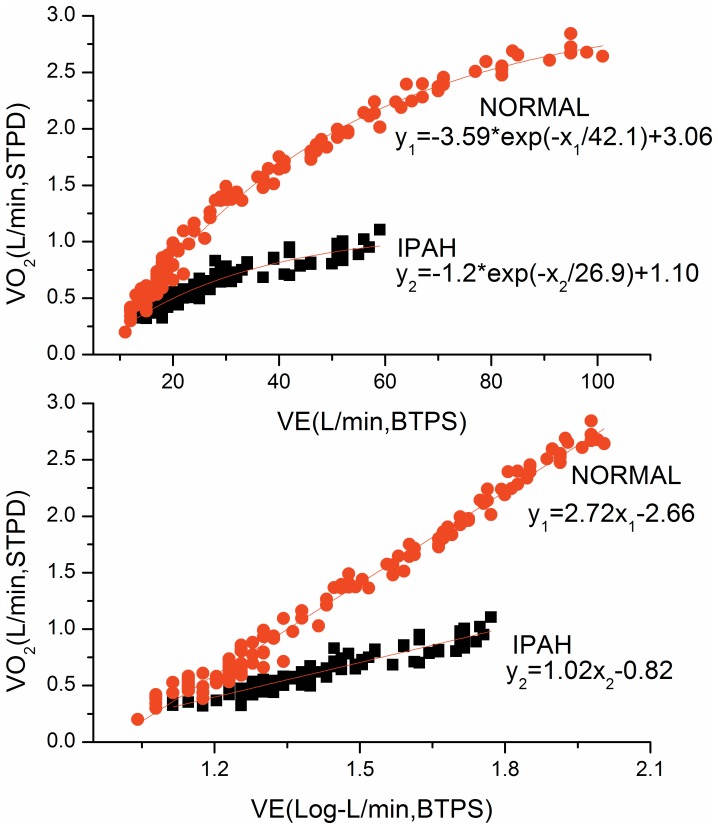
Difference of OUES between a typical IPAH patient and a control subject. Linear (upper panel) and single-segment logarithmic (lower panel) relation between V˙O_2_ (ml/min) and V˙E (ml/min) for 2 different subjects. Steeper slopes represents more efficient oxygen uptake. The control subject (steeper slopes, aged 24 years; height, 158 cm; weight, 45 kg), has an oxygen uptake efficiency slope (OUES) of 2.72 whereas the IPAH patient (shallower slopes, aged 21 years; height, 161 cm; weight, 47 kg) has an OUES of 1.02.

**Figure 2:**
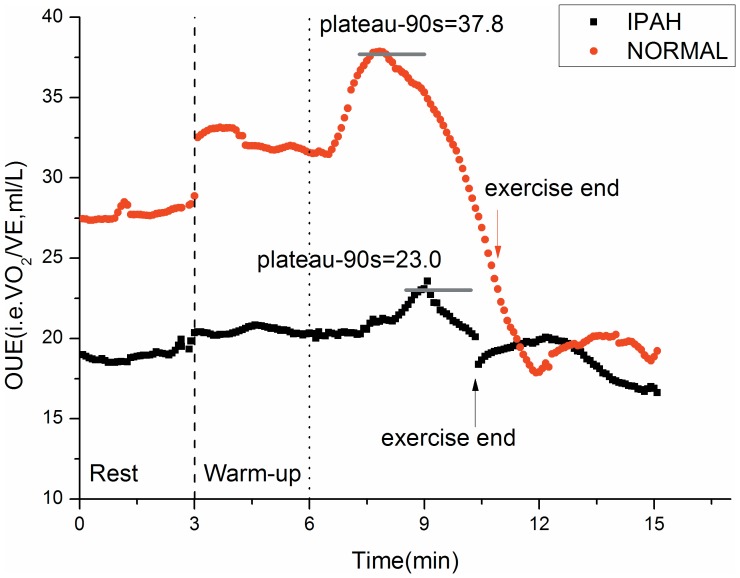
Difference of OUEP and OUE between a typical IPAH patient and a control subject. The kinetics of changes in oxygen uptake efficiency (OUE) for the same tests and subjects as depicted in Figure 1. OUE typically increase during exercise from rest to plateau in normal subjects and then decrease gradually until exercise end. It then decreases further in the immediate recovery period and begin stabilizing after about 2 minutes. In IPAH patients, OUE changes in a similar way as the controls, but is always lower than the controls in the transition from rest to exercise end.

The OUES was defined as the regression slope “a” in V˙O_2_ = a×log_10_V˙E+b. A steeper slope or higher OUES represents a more efficient oxygen uptake per volume of ventilation (Figure 1). The OUEP was defined as the 90 sec average of the highest consecutive measurements of V˙O_2_/V˙E near the AT (Figure 2) [14].

### Statistical analysis

Microsoft Office-2000, SPSS-10.0 and Origin-7.0 computer software were used. Data are expressed as mean ±SD, except where specifically noted. Most PFT and CPET values are expressed in absolute terms and %pred. *P*<0.05 was considered significant. Unpaired Student *t*-test was used for comparison between IPAH patients and normal subjects, whereas *X^2^* test was performed for gender analysis. The differences in OUE, V˙E/V˙CO_2_ and P_ET_CO_2_ at each time period were respectively assessed by repeated-measures analysis of variance (ANOVA). Correlations between OUE and other variables were determined by Pearson's correlation test, except for NYHA functional classification by Spearman rank correlation test.

## Results

### Baseline clinical and demographic characteristics

Characteristics of patients and healthy subjects are detailed in Table 1. The female-to-male ratio of the IPAH patients and healthy subjects in this study were about 2∶1. The PFT and CPET parameters of the healthy group were within normal limits. The DL_CO_ values were significantly lower in the IPAH patients compared with the normals. The FEV_1_/FVC in the IPAH group was significantly lower than the control group, but still within normal limit. Other PFT values were normal. 69% of IPAH patients were NYHA functional class 2 while 75% had cardiac index below 2.5.

**Table 1:** Demographics, hemodynamics, Pulmonary Function Testing and Cardiopulmonary Exercise Testing parameters in IPAH patients and Control subjects.

	IPAH patients (n = 32)	Control subjects (n = 16)
Age, yrs	40.3±14.8	37.9±16.8
Gender, F/M	20/12	10/6
Height, cm	162±7.8	160±9.0
Weight, kg	60.3±13.7	53.2±9.9
Body mass index, kg/m^2^	22.8±3.9	20.7±2.2
NYHA functional class	2.3±0.48	NA
mPAP, mm Hg	59.0±14.2	NA
mRAP, mm Hg	11.2±4.8	NA
mPWP, mmHg	8.6±4.4	NA
TPVR, mm Hg/L/min	13.1±5.7	NA
Cardiac index, L/min/m^2^	2.48±0.85	NA
FVC, L (%pred)	3.30±0.85 (95±20)	3.45±0.84 (100±14)
FEV_1_, L (%pred)	2.56±0.61(87±17)	2.93±0.62(101±15)
FEV_1_/FVC (%pred)	78.1±6.7(96±5) *	85.6±5.9(101±6)
MVV, L/min (%pred)	86±25 (98±19)	98±30 (116±18)
DL_CO_, ml/mm Hg/min (%pred)	17.1±6.5 (79±23) ‡	23.9±5.2(117±15)
TLC, L (%pred)	5.14±0.92 (99±11)	5.13±1.17 (100±12)
Peak V˙O_2_, ml/min (%pred)	920±298(49±14) ‡	1617±547 (95±15)
Peak work rate, W (%pred)	72±26(54±16) ‡	137±52(102±26)
AT, ml/min (%pred)	615±165(76±14) ‡	937±255(111±10)
Peak heart rate, beats/min (%pred)	146±17(80±7) †	166±12(90±7)
Peak O_2_ pulse, ml/beat (%pred)	6.2±1.7(63±17) ‡	9.6±2.8(97±7)
Peak V˙E, L/min (%MVV)	49±13(59±16)	61±23(62±7)
Peak P_ET_CO_2_, mm Hg	23.2±8.0‡	40.9±2.9
V˙E-V˙CO_2_ slope	51.7±28.1*	27.9±5.9
Lowest V˙E/V˙CO_2_, (%pred)	49.4±14.9(183±49) ‡	27.7±2.2(106±9)
OUES, L/min/log(L/min) (%pred)	1.08±0.37(58±19) ‡	1.98±0.44(98±13)
OUEP, ml/L (%pred)	23.4±4.9(60±12) ‡	37.8±4.8(98±12)

Values are expressed as mean ± SD and percentage of measured to predicted values (%pred).

*p<0.05, †*p*<0.005, ‡*p*<0.0001, vs. controls using unpaired *t* test. NA = not applicable.

NYHA  =  New York Heart Association functional classification; mPAP  =  mean pulmonary artery pressure; mRAP = mean right atrial pressure; mPWP = mean pulmonary artery wedge pressure; TPVR  =  total pulmonary vascular resistance; FVC  =  forced vital capacity; FEV_1_  =  forced expiratory volume in 1 second; MVV  =  maximum voluntary ventilation; DL_CO_  =  gas transfer index or diffusing capacity for carbon monoxide; TLC  =  total lung capacity; AT  =  anaerobic threshold; %MVV  =  percentage of maximum voluntary ventilation; P_ET_CO_2_  =  partial pressure of end-tidal carbon dioxide; OUES  =  oxygen uptake efficiency slope; OUEP  =  oxygen uptake efficiency plateau; IPAH  =  idiopathic pulmonary arterial hypertension; %pred  =  percent of predicted; V˙O_2_  =  peak oxygen uptake, STPD  =  standard temperature pressure dry; V˙E  =  minute ventilation, BTPS  =  body temperature pressure saturated; V˙CO_2_  =  carbon dioxide output, STPD.

All individuals completed their CPET studies without accident or untoward effects. Nearly all patients stopped exercise because of fatigue and/or acute shortness of breath; uncommonly, patients noted palpitations or light-headedness and recovered after resting for several minutes. All subjects declared they had done their best. In IPAH group, except for peak heart rate and peak ventilation, the magnitude of the absolute and percentage of all CPET parameters of oxygen uptake and ventilatory efficiency were strikingly abnormal.

### Decrease of OUES in IPAH

As shown in Figure 1, the typical case of IPAH had a lower OUES than the matched normal subject. The OUES of IPAH group was 1.08±0.37 which was significantly lower than 1.98±0.44 of control (p<0.0001).

### Changes and contributions in OUE and V˙E/V˙CO2 during CPET

As shown in Figure 2, the OUE response of IPAH patient to exercise was clearly different from that of the matched normal subject. At all times, the OUE values of IPAH patient were lower than those of control.

**Figure 3:**
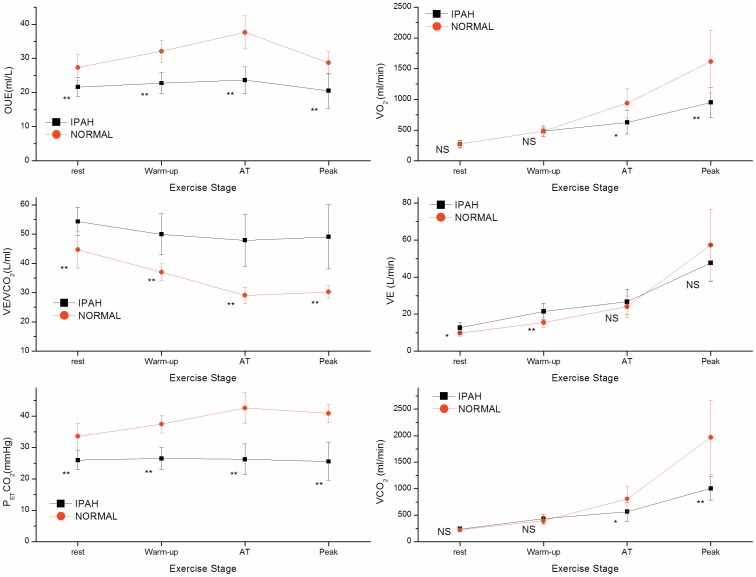
Difference of CPET parameters between IPAH and control groups at different stages of exercise. The group mean±SD Values of IPAH) and control (NORMAL) groups are shown at stages of rest, unloaded cycling, AT, and peak exercise during incremental cycle ergometry tests. Values are. On the left side from top to bottom, they are OUE, V˙E/V˙CO_2_ and P_ET_CO_2_.; on the right side from top to bottom they are V˙O_2_, V˙CO_2_ and V˙E. Statistically significant differences between groups at the same stage are shown as NS for no significance, * for P<0.05, ** for *P*<0.005, below value symbol.

The left top portion of Figure 3 shows OUE values for patients and normal subjects at four time periods. OUE values at all time periods were markedly lower in IPAH patients than in normal subjects (*P*<0.001). In control group, the differences in OUE values at four time periods were significant (*P*<0.001), and changes between adjacent periods were evident (*P*<0.001). However in IPAH group, the differences in OUE values at four time periods were also significant (*P* = 0.023), but the magnitude of OUE changes was much smaller than the control group.

Figure 3 left center, shows V˙E/V˙CO_2_ values at similar times. IPAH patients had significantly greater V˙E/V˙CO_2_ than normal subjects at all activity levels (*P*<0.001). From rest to the AT, V˙E/V˙CO_2_ values in control group reduced greatly, however in IPAH group decreased hardly. Compared with V˙E/V˙CO_2_ values at AT, there was no obvious reduction at peak either for controls or patients.

Figure 3 left bottom, shows the significantly reduced P_ET_CO_2_ values at all levels of activity in the IPAH group compared with the control group (*P*<0.001). In the control group, P_ET_CO_2_ values distinctively increased with increasing level of activity until AT, thereafter decreased mildly at peak. On the contrary, P_ET_CO_2_ values in IPAH patients did not increase at all from resting values.

Figure 3 right side, shows the similarities of both IPAH and Control groups for V˙O_2_ and V˙CO_2_ at rest and warm-up (*P*>0.05). There were significant differences at AT (*P*<0.05) and peak exercise (*P*<0.001). However, for V˙E, IPAH patients had higher values than those of Control subjects at rest (*P*<0.05) and warm-up (*P*<0.001), but no difference at AT and peak exercise (*P*>0.05). This indicates that at any required metabolic rate (as V˙O_2_ and V˙CO_2_), the ventilation is over driven by lung compensation for a limited heart function, i.e. mismatched Q/VA. The low and unchanged P_ET_CO_2_ is the evidence of hyperventilation in patients with IPAH.

### OUE as related to key abnormal parameters for IPAH patients

The correlations between OUE and other key parameters for IPAH patients are shown in Table 2. OUEP %pred correlated significantly with NYHA functional Class (*r* = −0.724, *P*<0.005), Total Pulmonary Vascular Resistance (TPVR) (*r* = −0.694, *P*<0.005), DLco %pred (*r* = 0.577, *P*<0.05), peak P_ET_CO_2_ (*r* = 0.68, *P*<0.005), and lowest V˙E/V˙CO_2_ (*r* = −0.902, *P*<0.0001). In contrast, the OUES %pred did not correlate significantly with above parameters (*r* = 0.125, −0.015, 0.493, 0.179, −0.136, all *P*>0.05).

**Table 2:** Correlations between OUE and key abnormal parameters for IPAH patients (N = 32).

	OUEP %pred	OUES %pred
NYHA	−0.724**	0.125
mPAP, mm Hg	−0.338	−0.351
TPVR, mm Hg/L/min	−0.694**	−0.015
CI, L/min/m^2^	0.295	0.047
DLco, ml/mm Hg/min	0.577*	0.493
PeakV˙O_2_, (%pred)	0.460	0.009
Peak P_ET_CO_2_, mm Hg	0.680**	0.179
Lowest V˙E/V˙CO_2_ (%pred)	−0.902**	−0.136

*P<0.05, **P<0.005.

The abbreviation definitions are same as Table 1.

### Comparison between OUEP and OUES for IPAH patients

Table 3 compares the mean and variability of the OUEP and OUES values in the 32 IPAH patients. The coefficient of variation(COV) of the OUEP (20.9%) was significantly lower than that of the OUES (34.3%) (*P*<0.0001).

**Table 3:** Mean, SD, range and COV of OUE measurements during Cardiopulmonary Exercise Testing in IPAH patients (N = 32).

	OUEP (ml/L)	OUES [L/min/log(L/min)]
Mean±SD	23.4±4.9	1.1±0.4
Range	11.0-31.0	0.51-1.76
COV	20.9%***	34.3%

***P<0.0001 by paired t test, versus OUES.

COV  =  coefficient of variation (SD/mean); all other abbreviation definitions are same as Table 1.

## Discussion

Although previous studies have demonstrated the clinical utility of CPET in patients with IPAH [3], our study is the first to evaluate the value of OUE measurements driven from CPET in these patients. In addition, our study is the first to show that the decreased OUE can also be a marker representing impaired gas exchange in patients with IPAH. Moreover, we have shown that, beyond the traditional measurements of exercise capacity and ventilatory efficiency, OUEP is better than OUES, because it is less variable and is more significantly correlated with resting pulmonary hemodynamics in these patients.

In the present study, the usual parameters of exercise capacity and gas exchange (V˙peak O_2_, peak work rate, anaerobic threshold, peak heart rate, peak O_2_ pulse, peak P_ET_CO_2_, V˙E-V˙CO_2_ slope and lowest V˙E/V˙CO_2_) were all abnormal in the IPAH patients (Table 1). Peak V˙O_2_, anaerobic threshold, V˙E-V˙CO_2_ slope, lowest V˙E/V˙CO_2_, and P_ET_CO_2_ are the most commonly used clinical parameters for diagnostic and prognostic information [8], [15]. Peak V˙O_2_ is reduced in patients with higher total pulmonary vascular resistance and lower cardiac index and is highly correlated with the amount of functional pulmonary vascular bed [23]. However, it is strongly influenced by the patients' motivation and supervisors' subjective choice of ending test. In searching for more objective, reliable sub-maximal variables, anaerobic threshold (AT) has been tested. Although AT is significantly correlated with peak V˙O_2_ [24], it is often not easy to identify, as was the case with 5 of our patients. The AT is also subject to substantial inter-observer and intra-observer variability [25]. Recently, the values of V˙E/V˙CO_2_ during moderate exercise have been demonstrated as diagnostic and prognostic values in heart failure patients [26].The mechanism responsible for elevated V˙E/V˙CO_2_ in IPAH patients is considered to be multifactorial. In normal subjects, the ventilatory response (V˙E) is approximately linear with the CO_2_ output (V˙CO_2_) during exercise before ventilatory compensation point [22]. In IPAH patients, elevated V˙E/V˙CO_2_ levels manifest that the ventilation of underperfused alveoli causes an increase in dead space ventilation [4]. Increased V˙E/V˙CO_2_ levels have also been significantly correlated with decreased cardiac output, elevated pulmonary arterial pressures, decreased alveolar-capillary membrane conductance, and diminished heart rate variability [27]–[29]. In patients with severe IPAH, the V˙E/V˙CO_2_ ratio correlates significantly with pulmonary vascular resistance but not with mean pulmonary arterial pressure or cardiac index [30]. Additionally, both resting and peak exercise P_ET_CO_2_ values have prognostic value in patients with heart failure [31]–[32]. However P_ET_CO_2_ values are susceptible to multiple factors such as acute hyperventilation, increased dead space (due to emphysema or other lung diseases), or rapid shallow breathing patterns, all of which will reduce the P_ET_CO_2_ independently of cardiac function [3]. Compared with all of the above CPET variables, the analysis of OUE has been limited, especially in patients with IPAH.

Neither OUES nor OUEP is included in the traditional 9-panel plots [9]. However, they can be measured noninvasively without additional patient effort [4]–[6]. The OUE may have important prognostic value in exercise physiology in patients with chronic heart failure [15], [33]. Davies et al[33] assessed OUES in 243 patients with chronic heart failure and found that only OUES was identified as the sole significant independent prognostic variable in a multivariable model, compared with standard exercise variables. We calculated reference values for OUEP and found that OUEP was the best predictor of mortality (*P*<0.0001) in a study of patients with left heart failure, better than OUES or any other CPET variables. When combined with oscillatory breathing, the odds ratio for death in 6 months increased to 56.3[14]–[15].

The OUE during exercise in normal subjects is mainly impacted by several factors including cardiac output; alveolar and dead space ventilation; and the matching of the changes in cardiac output and pulmonary blood flow with the increase in alveolar ventilation [14]. We postulate that decreased OUE during CPET in IPAH patients in our present study might be due to an abnormally high pulmonary vascular resistance, leading to the greater right ventricular afterload and reducing cardiac output as well as pulmonary blood flow. The highest OUE usually occurs near AT in normal subjects, because at that time ventilation is often most efficient and matching of perfusion to ventilation is optimal. However, in IPAH patients the volume of pulmonary capillary bed is reduced and the distal pulmonary arteries lose their ability to dilate during exercise. The lower ratio of V˙O_2_ to V˙E during CPET may be predominantly attributed to the inability to improve ventilation/perfusion match and distribution of blood flow to the metabolizing muscles for IPAH patients. Furthermore, we found that OUEP was sub-maximal exercise parameter, better than the OUES, and did not require maximal exertion, so the OUEP might be more fitted for IPAH patients unable to perform maximal exercise test. As shown in figure 2 and Table 3 in our present study, the OUEP was relatively easier to visualize, recognize, calculate and had less variability than OUES. Our study demonstrated that OUEP %pred was correlated negatively with NYHA functional class (*r* = −0.724, *P*<0.005), TPVR (*r* = −0.694, *P*<0.005), and lowest V˙E/V˙CO_2_ (*r* = −0.902, *P*<0.0001) and positively with DLco %pred (*r* = 0.577, *P*<0.05) and peak P_ET_CO_2_ (*r* = 0.68, *P*<0.005). In contrast, the OUES did not significantly correlate with above parameters. We also demonstrated that OUEP had less variability and higher predictability than OUES for normal subjects regardless of the age, gender, or height[14].

Recently,we were the first one to investigate the full exercise response pattern, exercise physiology and predictions of oxygen uptake efficiency (OUE, i.e. V˙O_2_/V˙E, ml/L) and ventilatory efficiency of carbon dioxide elimination (V˙E/V˙CO_2_), their key measurements OUEP and the lowest V˙E/V˙CO_2_, in normal subjects[14], [22] and described their pathophysiological evidence and prognostic importance of early death, specifically as %pred, in patients with left ventricular heart failure[14]–[16]. We also identified that oscillatory breathing did not interfere with measurements of OUEP, OUE@AT, lowest V˙E/V˙CO_2_ and V˙E/V˙CO_2_@AT. However, the OUE response during exercise and the OUEP and OUE@AT were not investigated for IPAH patients. As shown in Figure 3 in our present study, both OUE and V˙E/V˙CO_2_ abnormalities indicate lower ventilatory efficiency of oxygen uptake and carbon dioxide elimination in IPAH patients. They result from the compensative over driven hyperventilation in order to maintain the required metabolic rate of V˙O_2_ and V˙CO_2_ mainly due to the limitation of blood flow perfusion, i.e. Q/VA mismatch. This is a similar mechanism as we previously described in patients with left ventricular heart failure and IPAH[4], [6], [14]–[16]. This gas exchange pathophysiology is more clear and easy understanding after we created the new theoretical system of “Holistic Integrative Physiology and Medicine”, which demonstrates the intra-coupling pulmonary and cardiovascular systems for the maintenance of metabolic homeostasis in whole body, and gas exchange measurement of CPET is one typical clinical example[34]–[35]. V˙E can be performed by lungs only, but V˙O_2_ and V˙CO_2_ gas exchange needs lung-heart to work in coordination. In primary cardiovascular diseases without the malfunction of other systems, the heart function is limited (as lower V˙O_2_) and the lungs will compensate with hyperventilation (as higher V˙E). Therefore in this regard, the OUEP may be advantageous in evaluating cardiovascular function and gas exchange abnormality for patients with IPAH.

### Study Limitations

It is a single center study with smaller sample size; a higher ratio of female distribution (F20/M12). So we plan to do a future investigation to retrospectively and prospectively analyze all IPAH patients from our center.

## Conclusion

In conclusion, the OUEP, which can be calculated from retrospective data could offers a new, objective and effort-independent method for evaluating the gas exchange abnormality in patients with IPAH.
